# Supplementary material for: Supertoroidal light pulses as electromagnetic skyrmions propagating in free space
Source: Nat Commun. 2021 Oct 8;12:5891. doi: 10.1038/s41467-021-26037-w (PMC8501108; doi:10.1038/s41467-021-26037-w)
Supplement: Supplementary file 1 — Supplementary Information [file 41467_2021_26037_MOESM1_ESM.pdf]

Supplementary Note 1:

Derivation of the electric field of fundamental toroidal pulse.

The TE-mode electric field of the fundamental toroidal pulse is given by:

$$E = -4if_0\sqrt{\frac{\mu_0}{\varepsilon_0}}\frac{r(q_1+q_2-2ict)}{\left[r^2+(q_1+i\tau)(q_2-i\sigma)\right]^3} \tag{A1}$$

Where $\tau = z-ct$, $\sigma = z+ct$, and $c = 1/\sqrt{\mu_0\varepsilon_0}$ is the speed of light. For the paraxial limit condition, $q_2 \gg q_1$, the electric field can be simplified as:

$$E = -4if_0\sqrt{\frac{\mu_0}{\varepsilon_0}}\frac{r(q_2-2ict)}{\left[r^2+(q_1+i\tau)(q_2-i\sigma)\right]^3} \tag{A2}$$

Considering the field is a very short propagating pulse at the speed of *c*, and $\tau = z-ct$ represents the local time, we can use the approximation of $z \doteq ct$ to evaluate the $\sigma = z+ct \doteq 2z \doteq 2ct$, then the electric field can be derived as:

$$\begin{aligned}
E &= -4if_0\sqrt{\frac{\mu_0}{\varepsilon_0}}\frac{r(q_2-i2z)}{\left[r^2+(q_1+i\tau)(q_2-i2z)\right]^3} \\
&= -4if_0\sqrt{\frac{\mu_0}{\varepsilon_0}}\frac{r}{(q_2-i2z)^2}\cdot\frac{1}{\left[\frac{r^2}{q_2-i2z}+(q_1+i\tau)\right]^3} \\
&= -4if_0\sqrt{\frac{\mu_0}{\varepsilon_0}}r\left(\frac{q_2+i2z}{4z^2+q_2^2}\right)^2\cdot\frac{1}{\left[q_1+\frac{q_2r^2}{4z^2+q_2^2}+i\left(\tau+\frac{2zr^2}{4z^2+q_2^2}\right)\right]^3}
\end{aligned} \tag{A3}$$

Here we define the notations of radius of curvature, *R*(*z*), and beam waist profile, *w*(*z*), as

$$R(z) = z\left[1+\left(\frac{z_0}{z}\right)^2\right] = \frac{4z^2+q_2^2}{4z} \tag{A4}$$

$$w^2(z) = w_0^2\left[1+\left(\frac{z}{z_0}\right)^2\right] = \frac{q_1}{2q_2}\left(4z^2+q_2^2\right) \tag{A5}$$

with the Rayleigh length and basic waist constant given by $z_0 = \frac{q_2}{2}$ and $w_0^2 = \frac{q_1q_2}{2} = q_1z_0$. Substitute these two notations and equations (A4) and (A5) into equation (A3), we can simplify electric field expression as:

$$E=-4if_0\sqrt{\frac{\mu_0}{\varepsilon_0}}r\left[\frac{q_2\left(1+i\frac{2z}{q_2}\right)}{\frac{2q_2}{q_1}w^2}\right]^2\cdot\frac{1}{\left[q_1\left(1+\frac{r^2}{2w^2}\right)+i\left(\tau+\frac{r^2}{2R}\right)\right]^3}$$

$$=-4if_0\sqrt{\frac{\mu_0}{\varepsilon_0}}r\left[\frac{w_0^2\left(1+i\frac{z}{z_0}\right)}{2z_0w^2}\right]^2\cdot\frac{1}{\left[q_1\left(1+\frac{r^2}{2w^2}\right)+i\left(\tau+\frac{r^2}{2R}\right)\right]^3} \tag{A6}$$

$$=-if_0\sqrt{\frac{\mu_0}{\varepsilon_0}}r\frac{w_0^4}{z_0^2w^4}\left(1+i\frac{z}{z_0}\right)^2\cdot\frac{1}{\left[q_1\left(1+\frac{r^2}{2w^2}\right)+i\left(\tau+\frac{r^2}{2R}\right)\right]^3}$$

Applying Taylor expansion of $\sqrt{1+x^2}\exp\left(i\tan^{-1}x\right)=1+ix+O\left(\frac{x^2}{\sqrt{2}}\right)$, i.e. the approximation of

$$1+ix\doteq\sqrt{1+x^2}\exp\left(i\tan^{-1}x\right) \tag{A7}$$

Applying equation (A7), the numerator term in equation (A6) can be rewritten as:

$$\left(1+i\frac{z}{z_0}\right)^2=\left\{\sqrt{1+\left(\frac{z}{z_0}\right)^2}\exp\left[i\cdot\tan^{-1}\left(\frac{z}{z_0}\right)\right]\right\}^2=\frac{w^2}{w_0^2}\exp\left[i2\phi(z)\right] \tag{A8}$$

Where the $\phi(z)=\tan^{-1}\left(\frac{z}{z_0}\right)$ is the Gouy phase. Substitute equation (A8) into (A6) to carry on the simplification:

$$E=-if_0\sqrt{\frac{\mu_0}{\varepsilon_0}}r\frac{w_0^2}{z_0^2w^2}\exp\left[i2\phi(z)\right]\cdot\frac{1}{\left[q_1\left(1+\frac{r^2}{2w^2}\right)+i\left(\tau+\frac{r^2}{2R}\right)\right]^3}$$

$$=-if_0\sqrt{\frac{\mu_0}{\varepsilon_0}}\frac{rw_0^2}{z_0^2w^2}\cdot\frac{\left[q_1\left(1+\frac{r^2}{2w^2}\right)-i\left(\tau+\frac{r^2}{2R}\right)\right]^3}{\left[\left(q_1\left(1+\frac{r^2}{2w^2}\right)\right)^2+\left(\tau+\frac{r^2}{2R}\right)^2\right]^3}\cdot\exp\left[i2\phi(z)\right] \tag{A9}$$

Define the notations of radially scaled local time, $T(\mathbf{r},\tau)$, as

$$T=\frac{-\left(\tau+\frac{r^2}{2R}\right)}{q_1\left(1+\frac{r^2}{2w^2}\right)}=\frac{c\left(t-\frac{z+r^2/2R}{c}\right)}{q_1\left(1+\frac{r^2}{2w^2}\right)} \tag{A10}$$

Substitute equation (A10) into (A9) to carry on the simplification:

$$E = -if_0\sqrt{\frac{\mu_0}{\varepsilon_0}}\frac{rw_0^2}{z_0^2 w^2}\cdot\frac{\left[q_1\left(1+\frac{r^2}{2w^2}\right)\right]^3\left(1+iT\right)^3}{\left[\left(q_1\left(1+\frac{r^2}{2w^2}\right)\right)^2\left(1+T^2\right)\right]^3}\cdot\exp\left[i2\phi(z)\right]$$
$$= -if_0\sqrt{\frac{\mu_0}{\varepsilon_0}}\frac{rw_0^2}{z_0^2 w^2}\cdot\frac{\left(1+iT\right)^3}{\left(q_1\left(1+\frac{r^2}{2w^2}\right)\right)^3\left(1+T^2\right)^3}\cdot\exp\left[i2\phi(z)\right] \tag{A11}$$

Applying the Taylor approximation of equation (A7) to simplify the numerator term in equation (A11), we get:

$$\left(1+iT\right)^3 \doteq \left[\sqrt{1+T^2}\exp\left(i\tan^{-1}T\right)\right]^3 = \left(1+T^2\right)^{3/2}\exp\left(i\cdot 3\tan^{-1}T\right) \tag{A12}$$

and carry on the simplification of the electric field expression:

$$E = -if_0\sqrt{\frac{\mu_0}{\varepsilon_0}}\frac{rw_0^2}{z_0^2 w^2}\cdot\frac{\left(1+T^2\right)^{3/2}\exp\left(i\cdot 3\tan^{-1}T\right)}{\left(q_1\left(1+\frac{r^2}{2w^2}\right)\right)^3\left(1+T^2\right)^3}\cdot\exp\left[i\phi(z)\right]$$
$$= -if_0\sqrt{\frac{\mu_0}{\varepsilon_0}}\frac{rw_0^2}{z_0^2 w^2}\frac{1}{\left(q_1\left(1+\frac{r^2}{2w^2}\right)\right)^3\left(1+T^2\right)^{3/2}}\cdot\exp\left\{i\left[3\tan^{-1}T+2\phi(z)\right]\right\} \tag{A13}$$

Define the notations of local-time amplitude, $A(T)$, and local-time wavenumber, $k(T)$, as

$$A(T) = -f_0\sqrt{\frac{\mu_0}{\varepsilon_0}}\frac{1}{q_1^3(T^2+1)^{3/2}} = \frac{-f_0\mu_0 c}{q_1^3(T^2+1)^{3/2}} \tag{A14}$$

$$k(T) = 3\tan^{-1}T \tag{A15}$$

Substitute equations (A14) and (A15) into (A13), the final closed-form amplitude-phase expression is given by:

$$E = i\frac{w_0^2 rA(T)}{z_0^2 w^2\left(1+\frac{r^2}{2w^2}\right)^3}\exp\left\{i[k(T)+2\phi(z)]\right\} \tag{A16}$$

where the amplitude and phase functions are both space-time non-separable.

Supplementary Note 2:

Derivation of the electric field of higher-order supertoroidal pulses.

We start from the scalar generating function in EDEPT method:

$$f = f_0 \frac{e^{-s/q_3}}{(q_1 + i\tau)(s + q_2)^{\alpha}} \tag{B1}$$

Where $s \equiv r^2/(q_1 + i\tau) - i\sigma$, $\tau = z - ct$, $\sigma = z + ct$, $r = \sqrt{x^2 + y^2}$, $c = 1/\sqrt{\mu_0\varepsilon_0}$ is the speed of light, the parameters q_1, q_2, q_3 are real positive with units of length, and the real dimensionless parameter α must satisfy $\alpha \geq 1$ in order for the electromagnetic pulse to fulfill finite energy. In conventional method, it always assumes that $q_3 \to \infty$ and $\alpha = 1$. Here we break the limit of the parameter α, that can be any real number no less than one in our new derivation. The scalar generating function is given by:

$$\begin{aligned} f &= f_0 \frac{1}{(q_1 + i\tau)\left(\dfrac{r^2}{q_1 + i\tau} - i\sigma + q_2\right)^{\alpha}} \\ &= f_0 \frac{(q_1 + i\tau)^{\alpha-1}}{\left[r^2 + (q_1 + i\tau)(q_2 - i\sigma)\right]^{\alpha}} \end{aligned} \tag{B2}$$

The doughnut-like pulse is derived under a curled vector Hertz potential $\mathbf{\Pi} = \nabla \times \hat{\mathbf{z}} f(\mathbf{r}, t)$ in cylindrical coordinate (r, θ, z), the TE-mode electromagnetic field can be generated from Hertz potential by

$$\begin{cases} \mathbf{E}(\mathbf{r}, t) = -\mu_0 \dfrac{\partial}{\partial t} \nabla \times \mathbf{\Pi} = \hat{\boldsymbol{\theta}} \mu_0 \partial_r \partial_t f \\ \mathbf{H}(\mathbf{r}, t) = \nabla \times (\nabla \times \mathbf{\Pi}) = \hat{\mathbf{r}} \partial_r \partial_z f + \hat{\mathbf{z}} \left(\partial_z^2 - \dfrac{1}{c^2} \partial_t^2 \right) f \end{cases} \tag{B3}$$

For this pulse solution, the electric field is purely azimuthally polarized, and the magnetic field is along the radial and longitudinal directions with no azimuthal component. The azimuthally polarized electric field is derived as:

$$\begin{aligned} E &= \mu_0 \partial_z \partial_t f = \mu_0 f_0 \partial_\rho \partial_t \frac{(q_1 + i\tau)^{\alpha-1}}{\left[r^2 + (q_1 + i\tau)(q_2 - i\sigma)\right]^{\alpha}} \\ &= \mu_0 f_0 \left\{ \frac{-2\alpha(\alpha+1) icr (q_1 + i\tau)^{\alpha-1} (q_1 + q_2 - 2ict)}{\left[r^2 + (q_1 + i\tau)(q_2 - i\sigma)\right]^{\alpha+2}} + \frac{2(\alpha-1)\alpha icr (q_1 + i\tau)^{\alpha-2}}{\left[r^2 + (q_1 + i\tau)(q_2 - i\sigma)\right]^{\alpha+1}} \right\} \\ &= f_0 \sqrt{\frac{\mu_0}{\varepsilon_0}} \left\{ \frac{-2\alpha(\alpha+1) ir (q_1 + i\tau)^{\alpha-1} (q_1 + q_2 - 2ict)}{\left[r^2 + (q_1 + i\tau)(q_2 - i\sigma)\right]^{\alpha+2}} + \frac{2(\alpha-1)\alpha ir (q_1 + i\tau)^{\alpha-2}}{\left[r^2 + (q_1 + i\tau)(q_2 - i\sigma)\right]^{\alpha+1}} \right\} \end{aligned} \tag{B4}$$

This equation (B4) is the expression of high-order flying doughnut pulse. Note that when $\alpha = 1$, the equation (B4) will be reduced into the expression of fundamental flying doughnut pulse as equation (A1). For the paraxial limit condition, $q_2 \gg q_1$, and neglecting extremely small value of the second term, the equation (D2) can be simplified as:

$$E=-2\alpha(\alpha+1)if_0\sqrt{\frac{\mu_0}{\varepsilon_0}}\frac{r(q_1+i\tau)^{\alpha-1}(q_2-2ict)}{\left[r^2+(q_1+i\tau)(q_2-i\sigma)\right]^{\alpha+2}} \tag{B5}$$

Considering the field is a very short propagating pulse at the speed of *c*, and $\tau=z-ct$ represents the local time, we can use the approximation of $z\doteq ct$ to evaluate the $\sigma=z+ct\doteq 2z$, then the electric field can be derived as:

$$\begin{aligned}
E&=-2\alpha(\alpha+1)if_0\sqrt{\frac{\mu_0}{\varepsilon_0}}\frac{r(q_1+i\tau)^{\alpha-1}(q_2-i2z)}{\left[r^2+(q_1+i\tau)(q_2-i2z)\right]^{\alpha+2}}\\
&=-2\alpha(\alpha+1)if_0\sqrt{\frac{\mu_0}{\varepsilon_0}}r(q_1+i\tau)^{\alpha-1}\frac{1}{(q_2-i2z)^{\alpha+1}}\frac{1}{\left[\frac{r^2}{q_2-i2z}+(q_1+i\tau)\right]^{\alpha+2}}\\
&=-2\alpha(\alpha+1)if_0\sqrt{\frac{\mu_0}{\varepsilon_0}}r(q_1+i\tau)^{\alpha-1}\left(\frac{q_2+i2z}{4z^2+q_2^2}\right)^{\alpha+1}\frac{1}{\left[q_1+\frac{r^2(q_2+i2z)}{4z^2+q_2^2}+i\tau\right]^{\alpha+2}}\\
&=-2\alpha(\alpha+1)if_0\sqrt{\frac{\mu_0}{\varepsilon_0}}r(q_1+i\tau)^{\alpha-1}\left(\frac{q_2+i2z}{4z^2+q_2^2}\right)^{\alpha+1}\frac{1}{\left[q_1+\frac{q_2r^2}{4z^2+q_2^2}+i\left(\tau+\frac{2zr^2}{4z^2+q_2^2}\right)\right]^{\alpha+2}}
\end{aligned} \tag{B6}$$

Here we define the notations of radius of curvature, *R*(*z*), and beam waist profile, *w*(*z*), as equations (A4) and (A5), with the Rayleigh length and basic waist constant given by $z_0=\frac{q_2}{2}$ and $w_0^2=\frac{q_1q_2}{2}=q_1z_0$. Substitute these two notations and equations (A4) and (A5) into equation (B6), we can simplify electric field expression as:

$$\begin{aligned}
E&=-2\alpha(\alpha+1)if_0\sqrt{\frac{\mu_0}{\varepsilon_0}}r(q_1+i\tau)^{\alpha-1}\left[\frac{q_2\left(1+i\frac{2z}{q_2}\right)}{\frac{2q_2}{q_1}w^2}\right]^{\alpha+1}\frac{1}{\left[q_1\left(1+\frac{r^2}{2w^2}\right)+i\left(\tau+\frac{r^2}{2R}\right)\right]^{\alpha+2}}\\
&=-2\alpha(\alpha+1)if_0\sqrt{\frac{\mu_0}{\varepsilon_0}}r(q_1+i\tau)^{\alpha-1}\left[\frac{w_0^2\left(1+i\frac{z}{z_0}\right)}{2z_0w^2}\right]^{\alpha+1}\frac{1}{\left[q_1\left(1+\frac{r^2}{2w^2}\right)+i\left(\tau+\frac{r^2}{2R}\right)\right]^{\alpha+2}}\\
&=-\frac{\alpha(\alpha+1)}{2^\alpha}if_0\sqrt{\frac{\mu_0}{\varepsilon_0}}r(q_1+i\tau)^{\alpha-1}\frac{w_0^{2(\alpha+1)}}{z_0^{\alpha+1}w^{2(\alpha+1)}}\left(1+i\frac{z}{z_0}\right)^{\alpha+1}\frac{1}{\left[q_1\left(1+\frac{r^2}{2w^2}\right)+i\left(\tau+\frac{r^2}{2R}\right)\right]^{\alpha+2}}
\end{aligned} \tag{B7}$$

Applying Taylor approximation of equation (A7), the numerator term in equation (B7) can be rewritten as:

$$\left(1+i\frac{z}{z_0}\right)^{\alpha+1}=\left\{\sqrt{1+\left(\frac{z}{z_0}\right)^2}\exp\left[i\cdot\tan^{-1}\left(\frac{z}{z_0}\right)\right]\right\}^{\alpha+1}=\frac{w^{\alpha+1}}{w_0^{\alpha+1}}\exp\left[i(\alpha+1)\phi(z)\right] \tag{B8}$$

Where the $\phi(z)=\tan^{-1}\left(\frac{z}{z_0}\right)$ is the Gouy phase. Substitute equation (B8) into (B7) to carry on the simplification:

$$\begin{aligned} E &= -\frac{\alpha(\alpha+1)}{2^{\alpha}} i f_0 \sqrt{\frac{\mu_0}{\varepsilon_0}} r(q_1+i\tau)^{\alpha-1} \frac{w_0^{2(\alpha+1)}}{z_0^{\alpha+1} w^{2(\alpha+1)}} \exp\left[i(\alpha+1)\phi(z)\right] \cdot \frac{1}{\left[q_1\left(1+\frac{r^2}{2w^2}\right)+i\left(\tau+\frac{r^2}{2R}\right)\right]^{\alpha+2}} \\ &= -\frac{\alpha(\alpha+1)}{2^{\alpha}} i f_0 \sqrt{\frac{\mu_0}{\varepsilon_0}} \frac{w_0^{\alpha+1} r(q_1+i\tau)^{\alpha-1}}{z_0^{\alpha+1} w^{\alpha+1}} \cdot \frac{\left[q_1\left(1+\frac{r^2}{2w^2}\right)-i\left(\tau+\frac{r^2}{2R}\right)\right]^{\alpha+2}}{\left[\left(q_1\left(1+\frac{r^2}{2w^2}\right)\right)^2+\left(\tau+\frac{r^2}{2R}\right)^2\right]^{\alpha+2}} \cdot \exp\left[i(\alpha+1)\phi(z)\right] \end{aligned} \tag{B9}$$

Define the notations of radially scaled local time, $T(\mathbf{r},\tau)$, as equation (A10), and substitute equation (A10) into (B9) to carry on the simplification:

$$\begin{aligned} E &= -\alpha(\alpha+1) i f_0 \sqrt{\frac{\mu_0}{\varepsilon_0}} \frac{r(q_1+i\tau)^{\alpha-1}}{2^{\alpha}\left(z^2+z_0^2\right)^{(\alpha+1)/2}} \cdot \frac{\left[q_1\left(1+\frac{r^2}{2w^2}\right)\right]^{\alpha+2}\left(1+iT\right)^{\alpha+2}}{\left[\left(q_1\left(1+\frac{r^2}{2w^2}\right)\right)^2\left(1+T^2\right)\right]^{\alpha+2}} \cdot \exp\left[i(\alpha+1)\phi(z)\right] \\ &= -\frac{\alpha(\alpha+1)}{2^{\alpha}} i f_0 \sqrt{\frac{\mu_0}{\varepsilon_0}} \frac{w_0^{\alpha+1} r(q_1+i\tau)^{\alpha-1}}{z_0^{\alpha+1} w^{\alpha+1}} \cdot \frac{\left(1+iT\right)^{\alpha+2}}{\left(q_1\left(1+\frac{r^2}{2w^2}\right)\right)^{\alpha+2}\left(1+T^2\right)^{\alpha+2}} \cdot \exp\left[i(\alpha+1)\phi(z)\right] \end{aligned} \tag{B10}$$

Applying the Taylor approximation of equation (A7) to simplify the numerator term in equation (B10), we get:

$$\left(1+iT\right)^{\alpha+2} \doteq \left[\sqrt{1+T^2}\exp\left(i\tan^{-1}T\right)\right]^{\alpha+2} = \left(1+T^2\right)^{(\alpha+2)/2}\exp\left[i\cdot(\alpha+2)\tan^{-1}T\right] \tag{B11}$$

To simplify the numerator term in equation (B11), we carry on the simplification of the electric field expression:

$$\begin{aligned} E &= -\frac{\alpha(\alpha+1)}{2^{\alpha}} i f_0 \sqrt{\frac{\mu_0}{\varepsilon_0}} \frac{w_0^{\alpha+1} r(q_1+i\tau)^{\alpha-1}}{z_0^{\alpha+1} w^{\alpha+1}} \cdot \frac{\left(1+T^2\right)^{(\alpha+2)/2}\exp\left[i\cdot(\alpha+2)\tan^{-1}T\right]}{\left(q_1\left(1+\frac{r^2}{2w^2}\right)\right)^{\alpha+2}\left(1+T^2\right)^{\alpha+2}} \cdot \exp\left[i(\alpha+1)\phi(z)\right] \\ &= -\frac{\alpha(\alpha+1)}{2^{\alpha}} i f_0 \sqrt{\frac{\mu_0}{\varepsilon_0}} \frac{w_0^{\alpha+1} r(q_1+i\tau)^{\alpha-1}}{z_0^{\alpha+1} w^{\alpha+1}\left(q_1\left(1+\frac{r^2}{2w^2}\right)\right)^{\alpha+2}\left(1+T^2\right)^{(\alpha+2)/2}} \cdot \exp\left\{i\left[(\alpha+2)\tan^{-1}T+(\alpha+1)\phi(z)\right]\right\} \end{aligned} \tag{B12}$$

The numerator term in equation (B12) can be further simplified by using the Taylor approximation of equation (A7) to separate the amplitude and phase terms as:

$$(q_1 + i\tau)^{\alpha-1} = \left[q_1 \left(1 + i\frac{\tau}{q_1} \right) \right]^{\alpha-1} = \left(q_1 \sqrt{1 + \left(\frac{\tau}{q_1} \right)^2} \exp\left[i \tan^{-1}\left(\frac{\tau}{q_1} \right) \right] \right)^{\alpha-1}$$
$$= \left(q_1^2 + \tau^2 \right)^{(\alpha-1)/2} \exp\left[i(\alpha-1)\tan^{-1}\left(\frac{\tau}{q_1} \right) \right] \tag{B13}$$

Substitute equation (B13) into (B12) to carry on the derivation:

$$E = -\frac{\alpha(\alpha+1)}{2^\alpha} i f_0 \sqrt{\frac{\mu_0}{\varepsilon_0}} \frac{w_0^{\alpha+1} r \left(q_1^2 + \tau^2 \right)^{(\alpha-1)/2}}{z_0^{\alpha+1} w^{\alpha+1} \left(q_1 \left(1 + \frac{r^2}{2w^2} \right) \right)^{\alpha+2} \left(1 + T^2 \right)^{(\alpha+2)/2}} \cdot \exp\left\{ i \left[(\alpha-1)\tan^{-1}\left(\frac{\tau}{q_1} \right) + (\alpha+2)\tan^{-1} T + (\alpha+1)\phi(z) \right] \right\} \tag{B14}$$

Define the notations of generalized local-time amplitude, $A_\alpha(\mathbf{r},\tau)$, and generalized local-time wavenumber, $k_\alpha(\mathbf{r},\tau)$, as

$$A_\alpha(\mathbf{r},\tau) = -f_0 \sqrt{\frac{\mu_0}{\varepsilon_0}} \frac{\left(q_1^2 + \tau^2 \right)^{(\alpha-1)/2}}{q_1^{\alpha+2} (T^2+1)^{(\alpha+2)/2}} = \frac{-f_0 \mu_0 c \left(q_1^2 + \tau^2 \right)^{(\alpha-1)/2}}{q_1^{\alpha+2} (T^2+1)^{(\alpha+2)/2}} \tag{B15}$$

$$k_\alpha(\mathbf{r},\tau) = (\alpha-1)\tan^{-1}\left(\frac{\tau}{q_1} \right) + (\alpha+2)\tan^{-1} T \tag{B16}$$

Note that when $\alpha = 1$, the generalized local-time amplitude, $A_\alpha(\mathbf{r},\tau)$, and generalized local-time wavevector, $k_\alpha(\mathbf{r},\tau)$, equations (B15) and (B16) will be reduced into the fundamental local-time amplitude, $A(T)$, and fundamental local-time wavevector, $k(T)$, equations (A14) and (A15). Substitute equations (B15) and (B16) into (B14), the final closed-form amplitude-phase expression is given by:

$$E = i \frac{\alpha(\alpha+1) w_0^{\alpha+1} r A_\alpha(\mathbf{r},\tau)}{2^\alpha z_0^{\alpha+1} w^{\alpha+1} \left(1 + \frac{r^2}{2w^2} \right)^{\alpha+2}} \exp\{ i[k_\alpha(\mathbf{r},\tau) + (\alpha+1)\phi(z)] \} \tag{B17}$$

Note that when $\alpha = 1$, the amplitude-phase expression of high-order flying doughnut pulse of equation (B17) will be reduced into the amplitude-phase expression of fundamental flying doughnut pulse as equation (A16). Also, when the index of α goes higher, the energy of the pulse would be smaller because $\frac{\alpha(\alpha+1)}{2^\alpha} \to 0$ when α goes to infinity, that is consistent with the meaning in the finite-energy assumption in the conventional EDEPT method.

Supplementary Note 3:

Derivation of the full electromagnetic field of supertoroidal pulses.

Based on the scalar generating function in EDEPT method as equation (B2), the doughnut-like pulse is derived under a curled vector Hertz potential $\mathbf{\Pi} = \nabla \times \mathbf{z} f(\mathbf{r}, t)$ in cylindrical coordinate (r, θ, z), the TE-mode electromagnetic field can be generated from Hertz potential by equation (B3), thus the expressions for the full electromagnetic field, including azimuthally polarized electric field, radial and longitudinal components of magnetic field, can be given as:

$$\begin{aligned} E_\theta &= \mu_0 \partial_z \partial_t f = \mu_0 f_0 \partial_\rho \partial_t \frac{(q_1 + i\tau)^{\alpha-1}}{\left[r^2 + (q_1 + i\tau)(q_2 - i\sigma)\right]^\alpha} \\ &= \mu_0 c f_0 \left\{ \frac{-2\alpha(\alpha+1) ir (q_1 + i\tau)^{\alpha-1}(q_1 + q_2 - 2ict)}{\left[r^2 + (q_1 + i\tau)(q_2 - i\sigma)\right]^{\alpha+2}} + \frac{2(\alpha-1)\alpha ir (q_1 + i\tau)^{\alpha-2}}{\left[r^2 + (q_1 + i\tau)(q_2 - i\sigma)\right]^{\alpha+1}} \right\} \end{aligned} \tag{C1}$$

$$\begin{aligned} H_r &= \partial_r \partial_z f = f_0 \partial_r \partial_z \frac{(q_1 + i\tau)^{\alpha-1}}{\left[r^2 + (q_1 + i\tau)(q_2 - i\sigma)\right]^\alpha} \\ &= f_0 \left\{ \frac{2\alpha(\alpha+1) ir (q_1 + i\tau)^{\alpha-1}(q_2 - q_1 - 2iz)}{\left[r^2 + (q_1 + i\tau)(q_2 - i\sigma)\right]^{\alpha+2}} - \frac{2(\alpha-1)\alpha ir (q_1 + i\tau)^{\alpha-2}}{\left[r^2 + (q_1 + i\tau)(q_2 - i\sigma)\right]^{\alpha+1}} \right\} \end{aligned} \tag{C2}$$

$$\begin{aligned} H_z &= \left(\partial_z^2 - \frac{1}{c^2}\partial_t^2\right) f = f_0 \left(\partial_z^2 - \frac{1}{c^2}\partial_t^2\right) \frac{(q_1 + i\tau)^{\alpha-1}}{\left[r^2 + (q_1 + i\tau)(q_2 - i\sigma)\right]^\alpha} \\ &= f_0 \left\{ \frac{-4\alpha (q_1 + i\tau)^{\alpha-1}\left[r^2 - \alpha(q_1 + i\tau)(q_2 - i\sigma)\right]}{\left[r^2 + (q_1 + i\tau)(q_2 - i\sigma)\right]^{\alpha+2}} - \frac{4(\alpha-1)\alpha (q_1 + i\tau)^{\alpha-2}(q_2 - i\sigma)}{\left[r^2 + (q_1 + i\tau)(q_2 - i\sigma)\right]^{\alpha+1}} \right\} \end{aligned} \tag{C3}$$

Note that the second terms in the curly brackets of all the equations (C1)-(C3) are always extremely small value. And when $\alpha = 1$, the electromagnetic field of high-order flying doughnut, equations (C1)-(C3), is reduced into the conventional expression for the fundamental flying doughnut:

$$E_\theta = -4 i f_0 \mu_0 c \frac{r(q_1 + q_2 - 2ict)}{\left[r^2 + (q_1 + i\tau)(q_2 - i\sigma)\right]^3} \tag{C4}$$

$$H_r = 4 i f_0 \frac{r(q_2 - q_1 - 2iz)}{\left[r^2 + (q_1 + i\tau)(q_2 - i\sigma)\right]^3} \tag{C5}$$

$$H_z = -4 f_0 \frac{r^2 - (q_1 + i\tau)(q_2 - i\sigma)}{\left[r^2 + (q_1 + i\tau)(q_2 - i\sigma)\right]^3} \tag{C6}$$

For the paraxial limit condition, $q_2 \gg q_1$, in the second term is extremely small and can be neglected in the curly bracket for all the equations (C1)-(C3), because the denominator is extremely larger than the numerator, and in the first term in the curly bracket for all the equations (C1) and (C2), the $q_1 + q_2$ and $q_2 - q_1$ can both be replaced by q_2, then the electromagnetic field can be simplified as:

$$E_\theta = -2\alpha(\alpha+1) i f_0 \mu_0 c \frac{r(q_1+i\tau)^{\alpha-1}(q_2-2ict)}{\left[r^2+(q_1+i\tau)(q_2-i\sigma)\right]^{\alpha+2}} \tag{C7}$$

$$H_r = 2\alpha(\alpha+1) i f_0 \frac{r(q_1+i\tau)^{\alpha-1}(q_2-2iz)}{\left[r^2+(q_1+i\tau)(q_2-i\sigma)\right]^{\alpha+2}} \tag{C8}$$

$$H_z = -4\alpha f_0 \frac{(q_1+i\tau)^{\alpha-1}\left[r^2-\alpha(q_1+i\tau)(q_2-i\sigma)\right]}{\left[r^2+(q_1+i\tau)(q_2-i\sigma)\right]^{\alpha+2}} \tag{C9}$$

Considering the field is a very short propagating pulse at the speed of c, and $\tau = z - ct$ represents the local time, we can use the approximation of $z \doteq ct$ to evaluate the $\sigma = z + ct \doteq 2z$, and based on the definitions of radius of curvature, $R(z)$, and beam waist profile, $w(z)$, Gouy phase, $\phi(z)$, radially scaled local time, $T(\mathbf{r},\tau)$, generalized local-time amplitude, $A_\alpha(\mathbf{r},\tau)$, and generalized local-time wavenumber, $k_\alpha(\mathbf{r},\tau)$, in the derivation of the last Supplementary Information, we can further simplify the transverse electromagnetic field, equations (C7) and (C8), as:

$$E_\theta = i\frac{\alpha(\alpha+1) w_0^{\alpha+1} r A_\alpha(\mathbf{r},\tau)}{2^\alpha z_0^{\alpha+1} w^{\alpha+1}\left(1+\frac{r^2}{2w^2}\right)^{\alpha+2}} \exp\{i[k_\alpha(\mathbf{r},\tau)+(\alpha+1)\phi(z)]\} \tag{C10}$$

$$H_r = -i\sqrt{\frac{\varepsilon_0}{\mu_0}}\frac{\alpha(\alpha+1) w_0^{\alpha+1} r A_\alpha(\mathbf{r},\tau)}{2^\alpha z_0^{\alpha+1} w^{\alpha+1}\left(1+\frac{r^2}{2w^2}\right)^{\alpha+2}} \exp\{i[k_\alpha(\mathbf{r},\tau)+(\alpha+1)\phi(z)]\} \tag{C11}$$

The transverse electromagnetic field can reach a more compact formation if we create a notation of the complex amplitude of $\mathcal{A}_\alpha = i\frac{\alpha(\alpha+1) w_0^{\alpha+1} r A_\alpha(\mathbf{r},\tau)}{2^\alpha z_0^{\alpha+1} w^{\alpha+1}\left(1+r^2/(2w^2)\right)^{\alpha+2}}$, the unified expression of the transverse field can be given as:

$$\boldsymbol{\Psi}_\perp = \begin{bmatrix}\mathbf{E}_\perp \\ \mathbf{H}_\perp\end{bmatrix} = \begin{bmatrix}\hat{\boldsymbol{\theta}} \\ \sqrt{\frac{\varepsilon_0}{\mu_0}}\hat{\mathbf{r}}\end{bmatrix} \mathcal{A}_\alpha \exp\{i[k_\alpha(\mathbf{r},\tau)+(\alpha+1)\phi(z)]\} \tag{C12}$$

Supplementary Note 4: Numerical verification of Maxwell's equations for supertoroidal pulses.

To verify the reliability of our approach, we compared the results of the original equations derived from EDEPT method, the local-time amplitude-phase formation, and the numerical results by directly solving Maxwell's equations. The results from these various calculations indeed show the same results:

	Original equations	Amplitude-phase formation	Numerical solution of COMSOL
α = 1	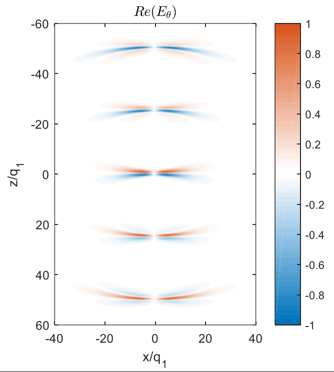	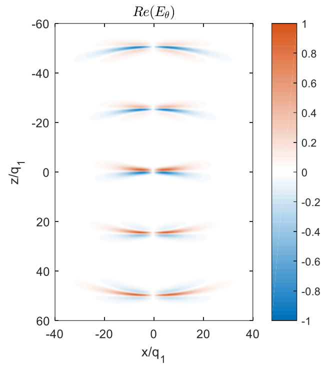	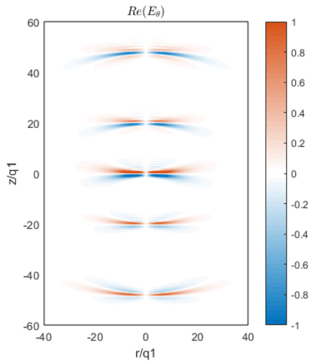
α = 3	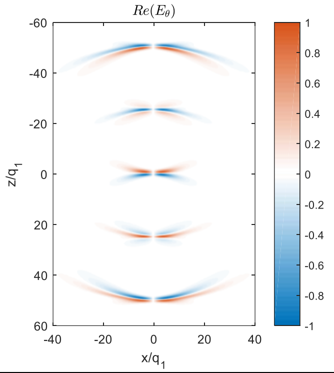	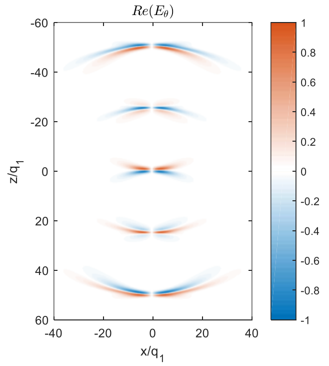	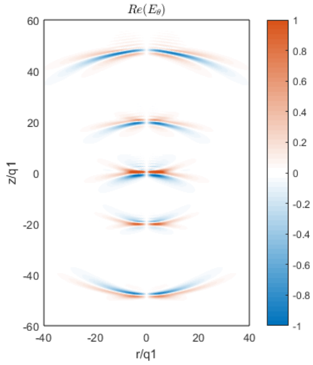
α = 6	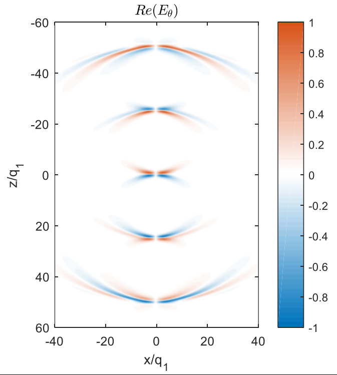	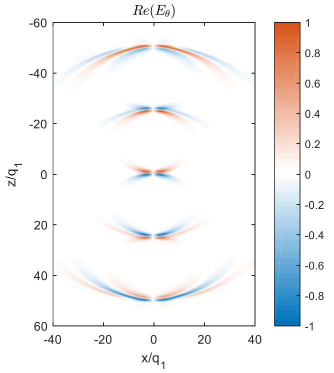	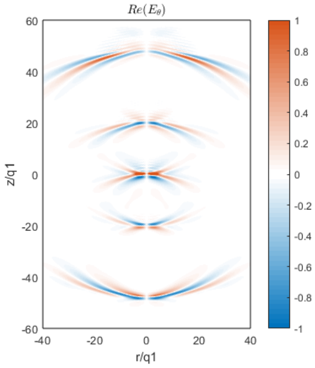
α = 10	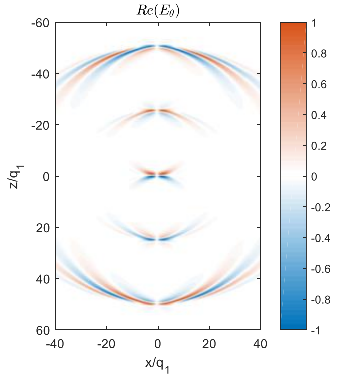	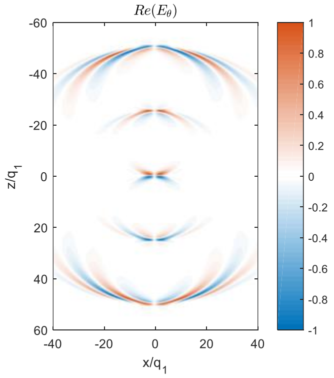	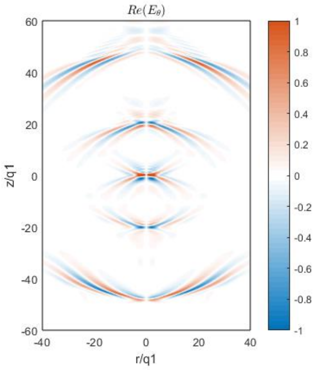

Supplementary Note 5: Spatial spectra of supertoroidal pulses.

Toroidal pulses are space-time non-separable solutions, and the space-time coupled structure can be revealed by the spatially dependent spectrum, as shown in the Fig. 1(a) of the spatial spectrum of a fundamental toroidal pulse at focus (q_2=100q_1, z=0), $\tilde{E}(r,\omega)$ that is the Fourier transformation of spatiotemporal function $E(r,t)$ at various radial positions. The supertoroidal pulses have increasingly complex space-time coupling due to the higher-order extension, some examples of which with various orders are shown in Fig. 1(b-f).

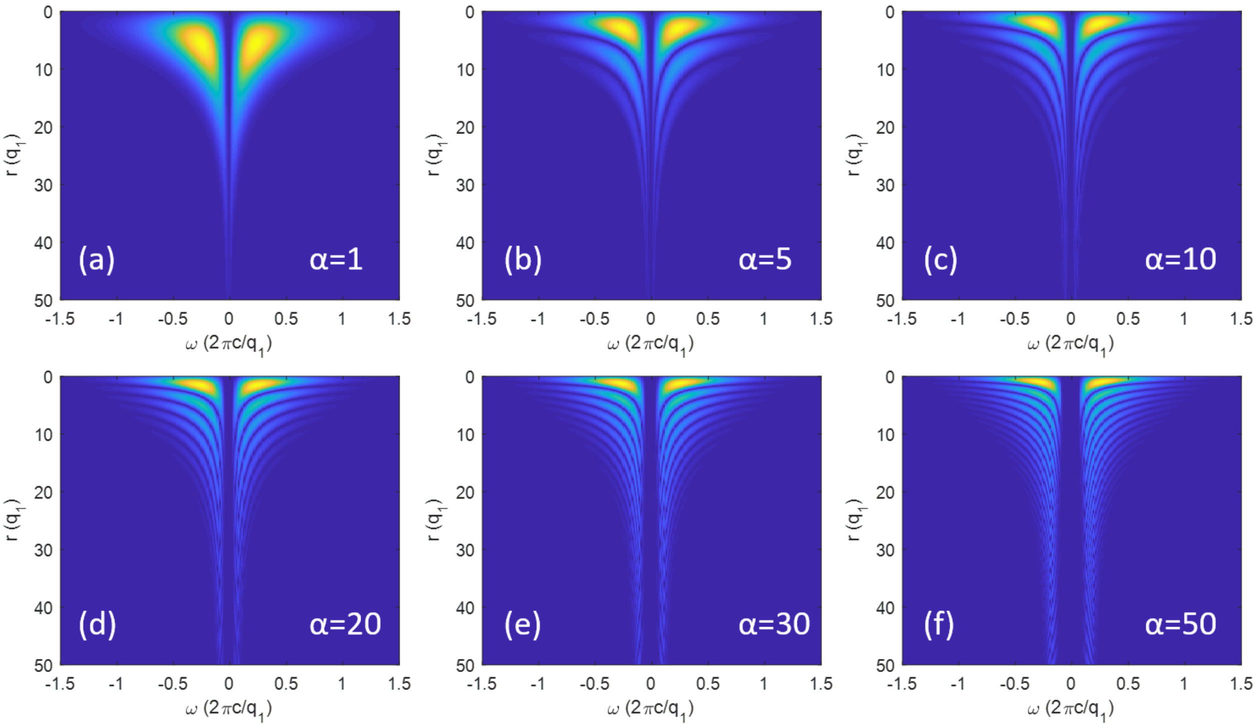

Fig. 1 (a-f) The theoretical results of spatio-spectral distributions of toroidal or supertoroidal pulses with different values of orders of α = 1, 5, 10, 20, 30, and 50, respectively.

Supplementary Note 6: Topological structures of skyrmions.

A skyrmion is a topologically stable 3D vector field configurations confined within a 2D domain, noted as $\mathbf{n}(x,y)$, which can be represented as the vector distribution unwrapped from the vector on a spiny sphere parametrized by longitude and latitude angles, *α* and *β*, see Fig. 2(a). As demonstrated in Methods, the topological properties of a skyrmionic configuration can be characterized by the skyrmion number:

$$\begin{aligned} s &= \frac{1}{4\pi}\iint_{\sigma}\mathbf{n}\cdot\left(\frac{\partial\mathbf{n}}{\partial x}\times\frac{\partial\mathbf{n}}{\partial y}\right)\mathrm{d}x\mathrm{d}y s = \frac{1}{4\pi}\int_0^{r_\sigma}\mathrm{d}r\int_0^{2\pi}\mathrm{d}\theta\frac{\mathrm{d}\beta(r)}{\mathrm{d}r}\frac{\mathrm{d}\alpha(\theta)}{\mathrm{d}\theta}\sin\beta(r) \\ &= \frac{1}{4\pi}[\cos\beta(r)]_{r=0}^{r=r_\sigma}[\alpha(\theta)]_{\theta=0}^{\theta=2\pi} = p\cdot m \end{aligned} \tag{F1}$$

where $\mathbf{n}(x,y)$ represents the vector field to construct a skyrmion and σ the region to confine the skyrmion, which can be infinity (for an isolated skyrmion) also can be a cell of a periodic distribution (for skyrmion lattices). The skyrmion number is an integer counting how many times the vector $\mathbf{n}(x,y)=\mathbf{n}(r\cos\theta, r\sin\theta)$ wraps around the unit sphere, as the mapping shown in Fig. 2(a). For mapping to the unit sphere, the vector can be given by $\mathbf{n}=(\cos\alpha(\theta)\sin\beta(r), \sin\alpha(\theta)\sin\beta(r), \cos\beta(r))$. The skyrmion number can be separated into two integers: the polarity, $p=\frac{1}{2}[\cos\beta(r)]_{r=0}^{r=r_\sigma}$, means that the vector direction is down (up) at center $r=0$ and up (down) at {boundary $r\rightarrow r_\sigma$ } for $p=1$ ($p=-1$), and the vorticity, $m=\frac{1}{2\pi}[\alpha(\theta)]_{\theta=0}^{\theta=2\pi}$, controls distribution of the transverse field components. In the case of a helical distribution, an initial phase γ should be added, $\alpha(\theta)=m\theta+\gamma$. Figures 2(b-g) show the theoretical results of selective topological structures of skyrmions with various values of polarity, vorticity, and helicity, (*p*, *m*, *γ*).

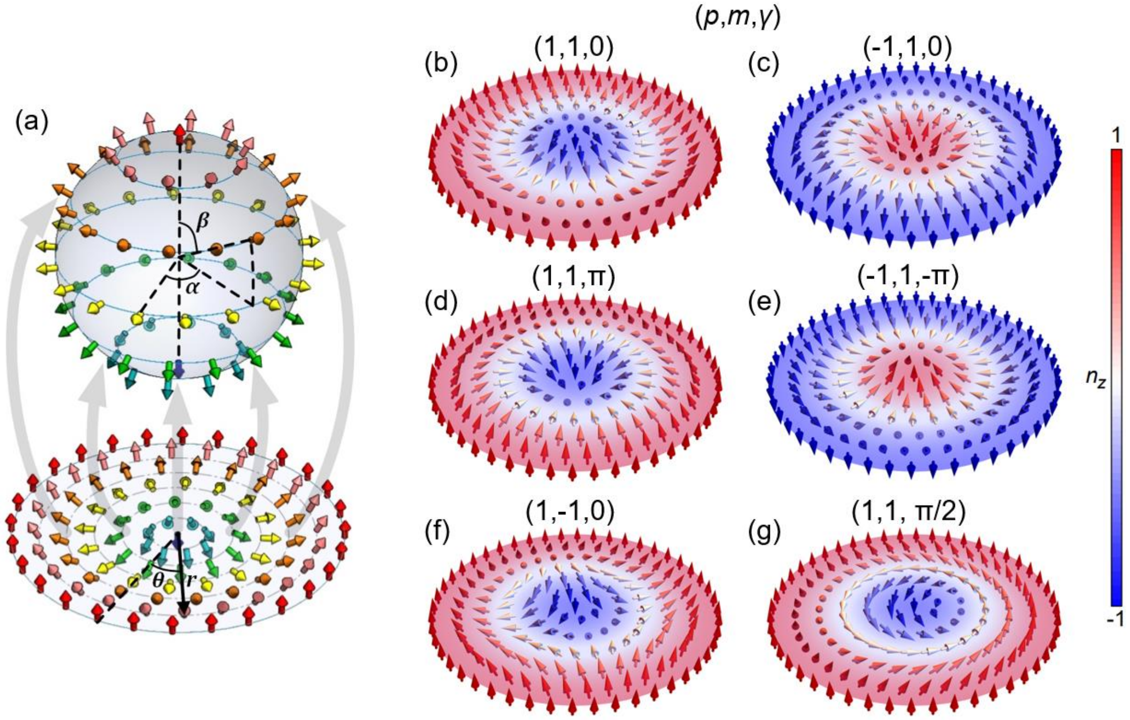

Fig. 2 (a) Representations of unit sphere wrapping for a skyrmion. (b-g) theoretical results of selective topological structures of skyrmions with various values of (*p*, *m*, *γ*) = (1,1,0), (-1,1,0), (1,1,π), (-1,1,-π), (1,-1,0), and (1,1, π/2), respectively.
