# Supplementary material for: Supertoroidal light pulses as electromagnetic skyrmions propagating in free space
Source: Nat Commun. 2021 Oct 8;12:5891. doi: 10.1038/s41467-021-26037-w (PMC8501108; doi:10.1038/s41467-021-26037-w)
Supplement: Supplementary file 3 — Description of Additional Supplementary Files [file 41467_2021_26037_MOESM3_ESM.docx]

**Description of Additional Supplementary Files**

File name: Supplementary Information

Description: Supplementary Information to support the results in the main text.

File name: Video 1

Description: Spatiotemporal dynamic evolution of fundamental toroidal light pulse.

File name: Video 2

Description: Spatiotemporal dynamic evolution of supertoroidal light pulse.

File name: Video 3

Description: Spatiotemporal distributions of (super)toroidal light pulses versus focus degree.

File name: Video 4

Description: Evolution of electromagnetic fields of supertoroidal light pulses versus supertoroidal index.

File name: Video 5

Description: Evolution of electromagnetic fields of supertoroidal light pulses upon propagating versus time.

File name: Video 6

Description: Evolution of electromagnetic fields of supertoroidal light pulses versus focus degree.
